# Glucosinolate variation, heterosis, and prediction of hybrid performance from parental values in white cabbage (*Brassica oleracea* var. *capitata*)

**DOI:** 10.3389/fpls.2026.1703515

**Published:** 2026-02-20

**Authors:** Primož Fabjan, Maja Mikulič-Petkovšek, Damijana Kastelec, Adriana Podržaj, Katarina Rudolf-Pilih

**Affiliations:** Biotechnical Faculty, University of Ljubljana, Ljubljana, Slovenia

**Keywords:** cabbage, doubled haploid lines, glucosinolates, heterosis, hybrid breeding, nutritional value

## Abstract

Glucosinolates (GSLs) are sulfur-containing secondary metabolites with important roles in plant defense and human health. Developing hybrids with increased GSL content is a promising approach to improve both resistance and health-related traits in cabbage. In other crops, moderately to highly heritable traits often show strong parent–hybrid correlations, enabling prediction of hybrid performance from parental phenotypes. Because GSLs show moderate to high heritability in closely related *Brassica* crops, this strategy has strong potential for developing cabbage hybrids with increased GSL content. Here, we evaluated GSL composition, variation, heterosis, and parent–hybrid correlations in 14 cabbage (*Brassica oleracea var. capitata*) doubled haploid lines, 11 derived hybrids, and two commercial hybrids. Desulfoglucosinolates were quantified by UHPLC–MS/MS using glucotropaeolin as an internal standard. Thirteen GSLs were detected across all genotypes, and total GSL concentrations ranged from 19.7 to 67.8 μmol g⁻¹ dw, indicating substantial genotype-dependent variation, with aliphatic GSLs dominating the profile (73.0%), followed by indolyl (26.9%) and aromatic (0.1%). Hierarchical clustering revealed two major genotype groups, differentiated primarily by 3C and 4C aliphatic GSLs, with correlation analysis reflecting coordinated regulation and trade-offs in their accumulation. Mid-parent heterosis (MPH) varied by compound and parental combination, with some GSLs showing consistently positive MPH (e.g., gluconapin) and others consistently negative MPH (e.g., neoglucobrassicin). Linear regression models showed that mid-parent values explained a high proportion of hybrid variability for major aliphatic glucosinolates and, to a lesser extent, for indolyl glucobrassicin, supporting the use of parental phenotyping to predict hybrid GSL performance. In parallel, agronomic traits showed uniformly positive mid-parent heterosis, with head weight exhibiting the highest values. Overall, these results support integrating parental GSL profiling into cabbage breeding to improve nutritional and defense-related traits alongside yield, thereby reducing the need for extensive testcrossing and field trials.

## 1 Introduction

Glucosinolates (GSLs) are sulfur- and nitrogen-rich secondary metabolites derived from amino acids. When plant tissue is damaged by pests or pathogens, GSLs are hydrolyzed by endogenous thioglucosidases (myrosinases), resulting in the formation of various degradation products (so-called “mustard oil bomb”), including isothiocyanates, thiocyanates, and nitriles (Prieto et al., 2019). These compounds play diverse biological roles in plants, particularly in responses to abiotic and biotic stresses, and are especially important in defense against herbivores and pathogens (Variyar et al., 2014). In recent years, GSLs and their breakdown products have received increasing attention because of their numerous health benefits, most notably their strong anticancer effects (Dinkova-Kostova and Kostov, 2012). On the other hand, some degradation products (particularly those derived from progoitrin) can interfere with thyroid function. However, these goitrogenic effects have only been observed in farm animals fed with oilseed rape meal, which contains exceedingly high concentrations of progoitrin (Cartea and Velasco, 2008).

A glucosinolate molecule consists of a β-D-glucopyranose moiety linked via a sulfur atom to a (Z)-cis-N-hydroximinosulfate ester and a variable side chain (R group) derived from amino acids. Glucosinolates can be classified based on the type of precursor amino acids: aliphatic glucosinolates are mainly derived from methionine (homo-methionine for 3-carbon (3C) aliphatic GSLs and dihomo-methionine for 4-carbon (4C) aliphatic GSLs), indolyl glucosinolates from tryptophan, and aromatic glucosinolates from phenylalanine and tyrosine. Their remarkable diversity in nature is mainly due to numerous modifications of the side chain, such as hydroxylation, O-methylation, acylation, glycosylation, and desaturation (Fahey et al., 2001).

To date, over 130 glucosinolates have been identified in nature, of which 88 have had their structures reliably confirmed using mass spectrometry (MS) and nuclear magnetic resonance (NMR) (Blažević et al., 2020). Glucosinolates are found almost exclusively in the order Brassicales, across approximately 4,700 species in 17 families. Among them, the mustard family (Brassicaceae) is the most agriculturally significant, as it includes many important vegetable crops and weeds (Agerbirk et al., 2008; Edger et al., 2018).

Cabbage (*Brassica oleracea* var. *capitata* L.) is one of the most widely cultivated vegetables in the Brassicaceae family. It is highly versatile and can be consumed in fresh salads, cooked, stir-fried, or eaten as a fermented product (Šamec et al., 2017). Cabbage consumption is associated with various health benefits, including anticancer, anti-inflammatory, and antioxidant effects, primarily attributed to its high glucosinolate and polyphenol content (Avato and Argentieri, 2015). As with many modern crops, most commercially grown cabbage varieties are hybrids because of their superior performance and uniformity. In recent years, plant breeding efforts have increasingly focused not only on improving yield, but also on enhancing resistance to different abiotic and biotic stresses, as well as improving nutritional value (Parkash et al., 2023). One promising approach to achieving these goals in cabbage is the development of varieties with elevated glucosinolate content, given their significant role in plant defense (Variyar et al., 2014) and their well-documented health benefits (Cartea and Velasco, 2008).

Owing to the presence of glucosinolates in the model species *Arabidopsis thaliana*, extensive knowledge has been accumulated on their biosynthetic pathways, regulatory networks, associated quantitative trait loci (QTL), and molecular markers (Harun et al., 2020). In cabbage, glucosinolate profiles typically comprise 13 compounds (**Table 1**), yet, despite the close phylogenetic relationship between *Arabidopsis* and cultivated *Brassica* crops, the genetic basis of glucosinolate variation in cabbage remains relatively underexplored. The recent annotation of the cabbage genome has enabled the identification of 193 genes involved in glucosinolate biosynthesis (Wang et al., 2023), providing a foundation for more detailed studies on their biosynthesis and regulation in this crop.

**Table 1:** Common, chemical names, their abbreviations, and structural classification of detected glucosinolates in cabbage genotypes, including the internal standard glucotropaeolin (GTP).

Chemical name (side chain)	Abbreviation	Common name	Class
3-methylthiopropyl	3mtp	Glucoiberverin (GBN)	Aliphatic 3C
3-methylsulfinylpropyl	3msp	Glucoiberin (GIB)	Aliphatic 3C
2-propenyl	2prop	Sinigrin (SIN)	Aliphatic 3C
2(R)-hydroxy-3-butenyl	2(R)-3but	Progoitrin (PRO)	Aliphatic 4C
2(S)-hydroxy-3-butenyl	2(S)-3but	Epiprogoitrin (EPRO)	Aliphatic 4C
3-butenyl	3but	Gluconapin (GNP)	Aliphatic 4C
4-methylsulfinylbutyl	4msb	Glucoraphanin (GRA)	Aliphatic 4C
4-methylthiobutyl	4mtb	Glucoerucin (GER)	Aliphatic 4C
indol-3-ylmethyl	I3M	Glucobrassicin (GBS)	Indolyl
4-methoxyindol-3-ylmethyl	4MOI3M	4-Methoxyglucobrassicin (MGBS)	Indolyl
4-methoxyindol-3-ylmethyl	4OHI3M	4-Hydroxyglucobrassicin (HGBS)	Indolyl
N-methoxyindol-3-ylmethyl	NMOI3M	Neoglucobrassicin (NGBS)	Indolyl
2-phenylethyl	2pe	Gluconasturtiin (GNS)	Aromatic
benzyl	benzyl	Glucotropaeolin (GTP)	Aromatic

Aliphatic 3-carbon (3C) GSLs are derived from homo-methionine, while aliphatic 4-carbon (4C) GSLs are derived from dihomo-methionine.

Research so far has mostly focused on glucosinolate content and profiles in different genotypes (Park et al., 2014; Bhandari et al., 2020), environmental condition (Cartea et al., 2008) and tissue types (Kabouw et al., 2010; Bhandari et al., 2015). To date, only three QTLs for glucosinolate content have been mapped in cabbage (Heon Oh et al., 2018), meaning that marker-assisted breeding for this trait is not yet feasible.

Studies in other crops, such as maize, have shown that traits with moderate to high heritability (e.g., grain-filling traits) exhibit significant heterosis and correlations between trait values of inbred lines and their derived hybrids (Alvarez Prado et al., 2013). Similar findings were reported in barley for grain yield, but with slightly lower values of heterosis and correlation coefficients (Mühleisen et al., 2013). This suggests that parental phenotypic values may serve as useful predictors of hybrid performance.

Given that glucosinolates show moderate to high heritability in closely related crops, such as *B. napus* (Ze-su et al., 2012) and *B. carinata* (Márquez-Lema et al., 2009), there is strong potential for this breeding strategy to be effective in developing cabbage hybrids with increased glucosinolate content. The objectives of this study were to: (i) explore the variation of glucosinolate content in cabbage lines and hybrids, (ii) evaluate the heterosis for glucosinolate content, and (iii) assess the relationship between glucosinolate content in parental lines and their derived hybrids.

## 2 Materials and methods

### 2.1 Plant material

Fourteen doubled haploid (DH) cabbage lines and eleven of their respective hybrids developed within our cabbage hybrid breeding program were evaluated in this study. Parental DH lines used for hybrid development were not selected randomly but were chosen to represent genetically diverse backgrounds, including lines derived from commercial hybrids and local landraces, in order to maximize genetic contrast relevant for hybrid breeding. Donor genotypes of DH lines are listed in **Supplementary Table S1**. The resulting hybrids included in this study comprise genotypes that were selected as promising candidate varieties based on multi-year field trials within the breeding program.

Inbred lines were designated numerical codes, while hybrids were denoted with the prefix “Hib” followed by a number. The maternal and paternal parent lines of hybrids are listed in parentheses in that order. Two commercial hybrids, Newton and Ambrosia, were included for comparison. DH lines were generated via microspore culture from heterozygous donor plants (**Supplementary Table S1**), following a previously optimized protocol (Rudolf Pilih et al., 2018), and maintained via micropropagation. Briefly, axillary buds were surface-sterilized using 1.6% dichloroisocyanuric acid, rinsed with sterile double-distilled water, and cultured on Murashige and Skoog (MS) medium containing 20 g L^−1^ sucrose, 8 g L^−1^ agar, 2 mg L^−1^ indolebutyric acid (IBA), and 3 mg L^−1^ benzylaminopurine (BAP). Shoots were regularly subcultured on the same medium, and rooting was induced on half-strength MS medium without growth regulators. Rooted plantlets were acclimatized in mini greenhouses before potting.

At the stage of 4–5 true leaves, six plants per genotype were transplanted into a greenhouse with drip irrigation. Controlled-release fertilizer was added at transplanting (Osmocote Exact, 15-9-12 + 2 MgO + TE). Registered pesticides were used as needed. At physiological maturity (mid-June to mid-July), four healthy heads per genotype were harvested, or three in cases where pest or disease damage was present.

From each head, approximately 10 g of leaf tissue was collected from the upper, central part of the head using a fixed-size template (4 x 4 cm) and excised to a constant depth of 1 cm. Sampling was standardized by tissue depth rather than by leaf number to account for genotype-dependent variation in leaf thickness. Samples were immediately frozen in liquid nitrogen and freeze-dried in a laboratory freeze dryer (CryoDryer 10, Augsburg, Germany) under the following conditions: primary drying at 0.5 mbar and –40 °C for 4 days, followed by secondary drying at 0.03 mbar and 30 °C. Freeze-dried samples were then ground in liquid nitrogen and stored at –80 °C.

Agronomic traits (head weight, diameter, height, inner core length, and compactness) were also recorded.

### 2.2 Glucosinolate analysis

#### 2.2.1 Chemicals

Authentic analytical glucosinolate standards were purchased from Cfm Oskar Tropitzsch (Marktredwitz, Germany) and Extrasynthese (Genay, France). According to the certificates provided by the manufacturers, the purity of the standards ranged from 95% to 99%, and corresponding HPLC chromatograms were supplied to confirm compound identity and purity. Acetonitrile (MS-grade) was purchased from J.T. Baker (Phillipsburg, NJ, USA). All other reagents (sulfatase (Type H-1), DEAE-Sephadex A-25, sodium acetate, methanol, and formic acid) were purchased from Sigma-Aldrich Chemie (Steinheim, Germany).

#### 2.2.2 Extraction and desulfation of glucosinolates

Glucosinolates were extracted from 50 mg of freeze-dried, powdered cabbage tissue using a modified method based on Ishida et al. (2011). Tissue was mixed with 3 mL 85% methanol containing 25 nmol glucotropaeolin (internal standard) in 5 mL tubes, sonicated at 130 W for 30 min (Sonis 4, Iskra Pio, Šentjernej, Slovenia), then shaken at 120 rpm for 30 min (Ecotron, Infors HT, Bottmingen, Switzerland). After centrifugation (2, 500 × g, 10 min), the supernatant was collected for desulfation according to a modified protocol from Doheny-Adams et al. (2017).

For desulfation, glass columns pre-filled with DEAE-Sephadex A-25 suspension were prepared as described by Grosser and van Dam (2017). Briefly, DEAE-Sephadex A-25 was suspended in double-distilled water (1g per 12.5 mL) and allowed to swell overnight at room temperature. Prior to the addition of DEAE-Sephadex A-25, a glass bead was placed into each glass column (Pasteur pipettes, Sigma-Aldrich Chemie, Steinheim, Germany) to prevent resin loss. A fixed volume (500 µL) of the equilibrated resin slurry was then transferred into each glass column, ensuring uniform slurry loading across all samples. The column material was then flushed down with 1 mL of double-distilled water.

Extracts were then loaded onto the columns. After discarding the flow-through, the columns were washed sequentially with 2 × 1 mL of 70% methanol, 1 mL of water, and 2 × 1 mL of 20 mM sodium acetate buffer (pH 5.5). Sulfatase solution (75 µL; 5 U/mL) was added to each column, and samples were incubated at room temperature for 24 h. Desulfoglucosinolates were eluted with 2 × 0.5 mL of double-distilled water, filtered through 0.22 µm cellulose acetate syringe filters (Macherey-Nagel, Düren, Germany), and stored at –80 °C until further analysis.

#### 2.2.3 UHPLC–MS/MS analysis of desulfoglucosinolates

Desulfoglucosinolates were quantified using ultra-high-performance liquid chromatography coupled with tandem mass spectrometry (UHPLC–MS/MS; TSQ Quantum Access MAX, Thermo Fisher Scientific, San Jose, CA, USA). Separation was performed on a Gemini C18 column (150 × 4.6 mm, 3 µm, 110 Å; Phenomenex, Torrance, CA, USA), maintained at 30 °C. The injection volume was 20 µL, and the flow rate was 0.4 ml min^-1^.

Mobile phase A consisted of 0.1% formic acid and 3% acetonitrile in water, while mobile phase B contained 0.1% formic acid and 3% water in acetonitrile. The gradient was adapted from Choi et al. (2014) and selected after testing several gradient profiles reported in literature (Crocoll et al., 2016; Grosser and van Dam, 2017; Bhandari et al., 2020). The Choi et al. (2014) gradient provided the best separation of desulfoglucosinolates and resulted in well-separated peaks. Formic acid was added to both mobile phases to improve ionization efficiency during MS detection, which is a standard practice in MS analysis (Crocoll et al., 2016). The final gradient was as follows: 0–5 min isocratic at 2% B; 5–45 min linear gradient to 45% B; and 45–47 min return to 2% B.

Mass spectrometric detection was performed using a heated electrospray ionization (HESI-II) source operating in positive ion mode. The full-scan range was set to m/z 90–700. Source parameters were as follows: spray voltage, 3.8 kV; capillary temperature, 320 °C; collision energy, 20 V; sheath gas pressure, 50 L h-1; and auxiliary gas pressure, 20 L h^-1^.

Data acquisition was performed in multiple reaction monitoring (MRM) mode. Each dsGSL was detected as its protonated molecular ion [M+H]^+^, and a characteristic fragment ion was used for quantification. MRM transitions were selected based on literature values (Crocoll et al., 2016; Singh et al., 2017) and validated using authentic standards. Peak integration was performed using Thermo Xcalibur software. Calibration curves for each analyte and the internal standard (IS) were constructed, and response factors (RFs) were calculated as the ratio of slopes:


$$RF=\;\frac{slope_{IS}}{slope_{analyte}}$$

The amount of each dsGSL was calculated relative to the internal standard using the equation from Crocoll et al. (2016):


$$nmol_{analyte}=(\frac{area_{analyte}}{area_{IS}})\times RF\times nmol_{IS}$$

The calculated values were normalized to the weight of the extracted material, and results were expressed as µmol per gram of dry weight (µmol g^−1^ dw).

### 2.3 Statistical analysis

Exploratory multivariate statistical analyses were conducted to characterize the average GSL composition of the selected genotypes. Because glucosinolate profiles constitute compositional data, hierarchical clustering was performed using the Aitchison distance following log-ratio transformation. Spearman correlation coefficients were used to identify patterns of association among individual GSL compounds. The results were visualized as a heatmap.

To assess differences in total GSL content between the two genotype groups (hybrids and lines), a two-sample t-test assuming independent observations was performed. For the individual GSL components, p-values were adjusted for familywise error rate using the Bonferroni correction. The same tests were made for agronomic traits.

Mid-parent heterosis (MPH) for individual and total glucosinolate content in 11 hybrids was calculated based on the formula:


$$MPH=\frac{\left(H-MPV\right)}{MPV}\times 100$$

MPV is the mid-parent value, calculated as the average of both parents, and H is the hybrid value for each glucosinolate.

Due to different variability and the presence of outliers in the mid-parent heterosis (MPH) data, nonparametric bootstrap resampling was used to estimate 95% confidence intervals (CIs) for the median MPH of each GSL. The bootstrap procedure does not rely on distributional assumptions and is therefore robust to deviations from normality and extreme values. Estimating CIs for the median provided a robust assessment of whether heterosis significantly differed from zero across hybrids. Bootstrap confidence intervals were calculated based on 1, 000 resamples.

The relationship between hybrid GSL concentrations and their respective mid-parent values (MPVs) was evaluated using linear regression models. Predictions from the models were generated with 95% confidence intervals for individual and total GSL content.

## 3 Results

### 3.1 Overview of glucosinolate content in cabbage genotypes

A total of 13 individual GSLs were detected across all cabbage genotypes, including eight aliphatic (PRO, EPRO, GNP, GRA, GER, SIN, GIB, and GBN), four indolyl (GBS, MGBS, HGBS, and NGBS), and one aromatic compound (GNS). **Table 1** summarizes each compound’s common and full chemical name, abbreviation, and class (including the internal standard).

**Figure 1:**
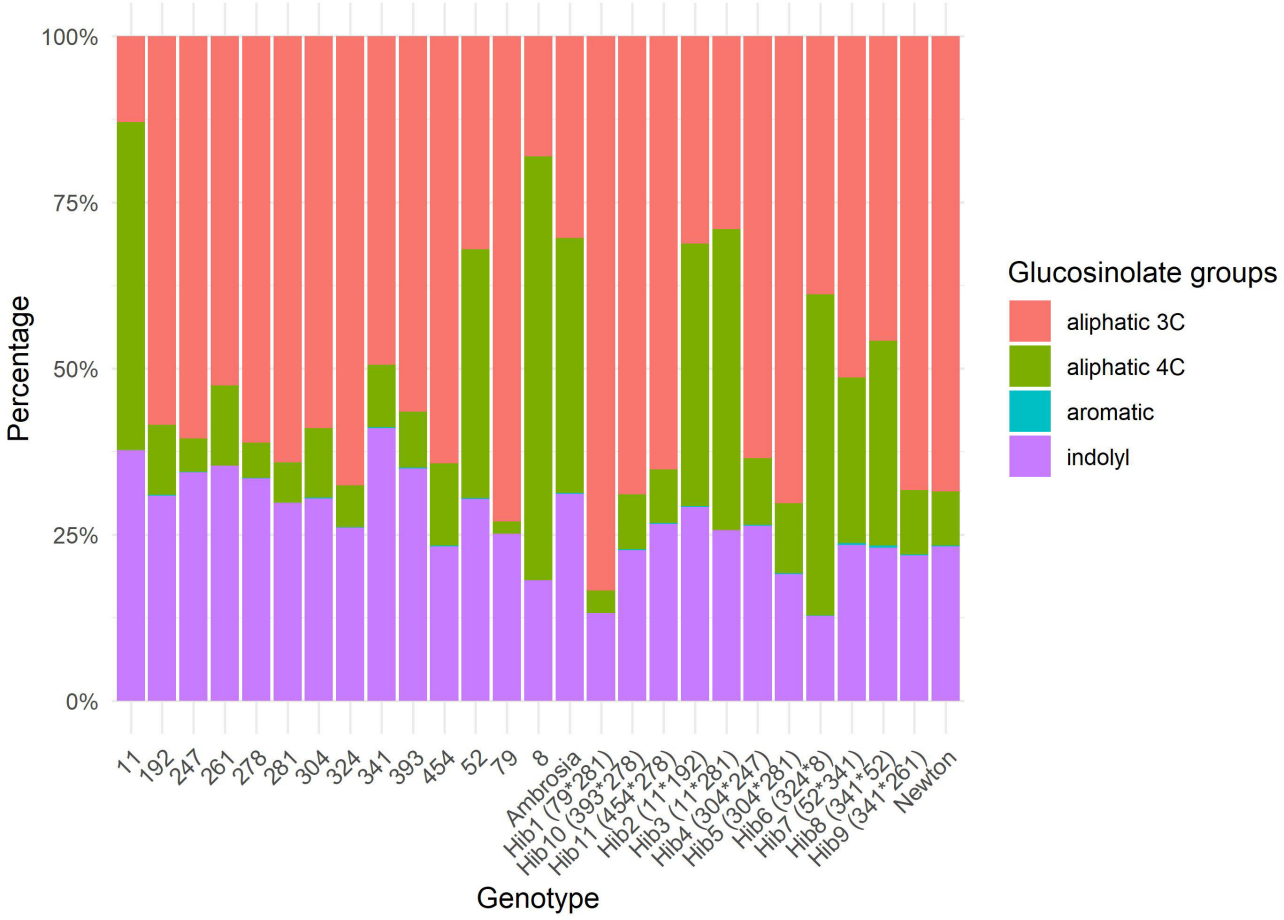
Mean proportional composition of glucosinolate classes (aliphatic 3C, aliphatic 4C, indolyl, and aromatic) in each cabbage genotype.

All GSLs were detected in each genotype except gluconasturtiin, which was absent in seven genotypes (lines 8, 11, 79, 261, 281, and hybrids 1 and 3). On average, aliphatic GSL accounted for 73.0% of the total GSL content, indolyl for 26.9%, and aromatic for only 0.1%. **Figure 1** shows the mean proportional composition of GSL classes (aliphatic 3C, aliphatic 4C, indolyl, and aromatic) in all genotypes. The detailed composition of individual GSLs in all genotypes is presented in **Supplementary Figure S1**.

**Figure 2:**
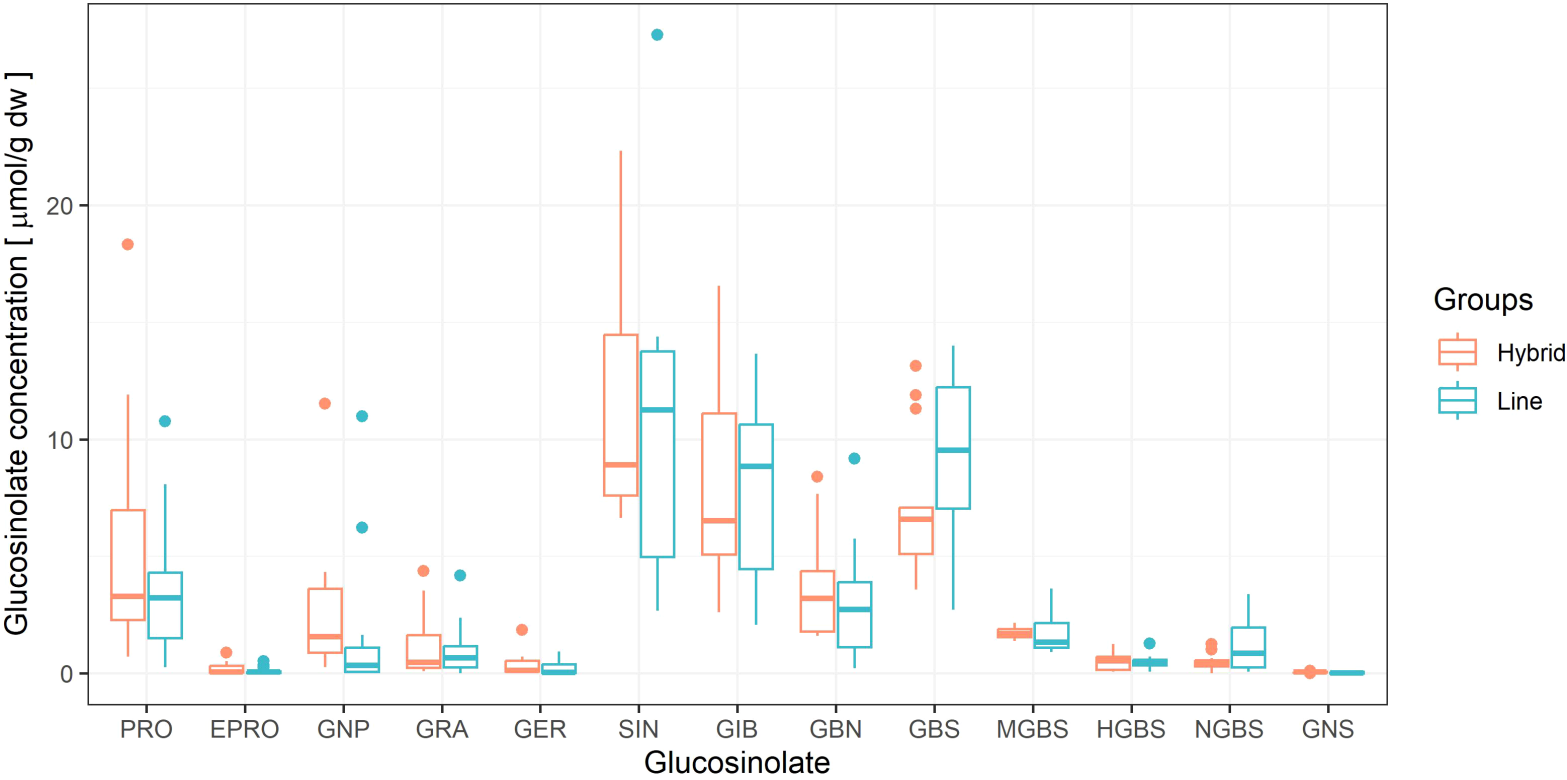
Boxplots of individual glucosinolate concentrations in cabbage hybrids (orange) and inbred lines (blue), showing median, interquartile range (Q1–Q3), and outliers.

Substantial variation in glucosinolate composition was observed among the 25 genotypes. Total mean glucosinolate concentration ranged from 19.7 µmol g^−1^ dw (line 324) to 67.8 μmol g^−1^ dw (line 281), with an overall mean of 42.0 μmol g^−1^ dw (**Supplementary Table S2**). No significant differences were found between genotype groups (lines vs. hybrids) for either individual or total glucosinolate concentrations (p > 0.05). This is also evident in **Figure 2**, which shows the variability of 13 individual glucosinolates in the two genotype groups.

Among the identified glucosinolates, five compounds (SIN, GBS, GIB, PRO, and GBN) were most abundant (**Figure 2**). Their mean concentrations across all genotypes were 11.1 ± 1.2, 8.2 ± 0.7, 7.9 ± 0.8, 4.7 ± 0.8, and 3.3 ± 0.5 µmol g^−1^ dw, respectively (**Supplementary Table S2**). Together they accounted for approximately 84% of the total glucosinolates. SIN was present at the highest concentrations, ranging from 2.68 to 27.28 µmol g^−1^ dw, and represented over 40% of the total glucosinolates in several genotypes, including hybrids 10, 8, and 4 (**Supplementary Figure S1**). GBS ranged from 2.7 to 14.06 µmol g^−1^ dw, GIB from 2.01 to 16.66 µmol g^−1^ dw, and PRO from 0.3 to 18.36 µmol g^−1^ dw. Among the aliphatic glucosinolates, EPRO and GER were the least abundant, together accounting for only 1.01% to the total glucosinolates. Among the indolyl glucosinolates, HGBS and NGBS were the least represented, together accounting for 3.3% of the total glucosinolate content. Mean concentrations with standard errors (three to four replicates per genotype) for all glucosinolates in individual and across all genotypes are provided in **Supplementary Table S2**.

To further explore the relationships among genotypes based on their glucosinolate profiles, hierarchical clustering was performed to reveal patterns and group genotypes with similar glucosinolate profiles. Based on correlation coefficients, the glucosinolates were also clustered into groups. The analysis was based on the mean concentrations of each of 13 glucosinolates across all genotypes.

**Figure 3:**
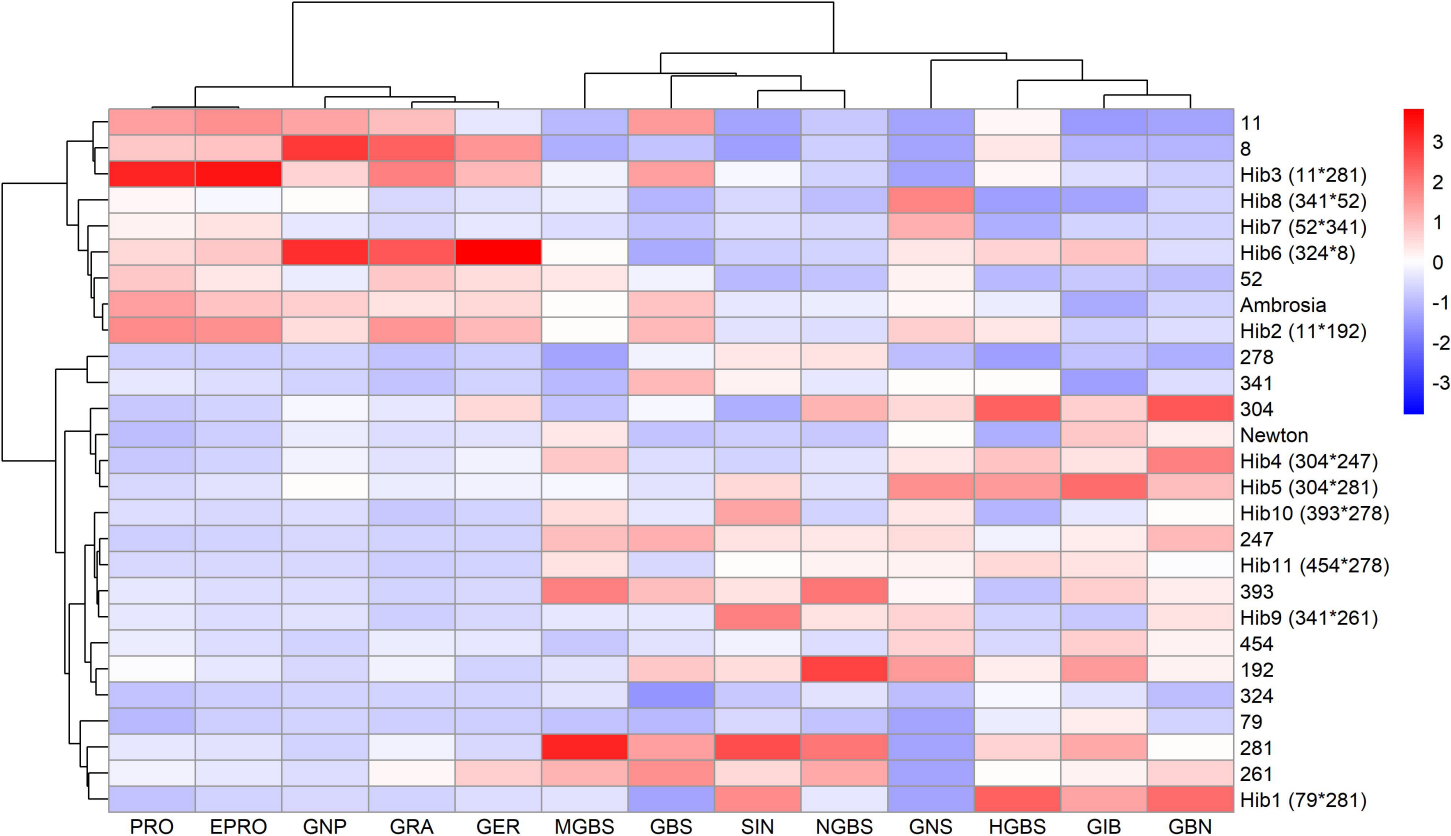
Heatmap of standardized glucosinolate content in all genotypes. Values above the mean are in red, and values below the mean are in blue. Hierarchical clustering was performed to group genotypes based on glucosinolate profiles. Glucosinolates were clustered based on correlation coefficients.

The resulting heatmap (**Figure 3**) revealed two major genotype clusters, primarily differentiated by aliphatic glucosinolates, which correspond to their higher overall concentrations, as discussed in the previous paragraph. The concentrations of the glucosinolates were presented as standardized values in **Figure 3**, so it can be quickly seen whether some glucosinolate content is higher (red) or lower (blue) than the mean for each genotype.

The first and smaller cluster consisted of genotypes with higher levels of 4-carbon (4C) aliphatic glucosinolates (PRO, EPRO, GNP, GRA, and GER) and lower levels of 3-carbon (3C) aliphatic glucosinolates (SIN, GBN, and GIB). A weaker trend was observed for some indolyl glucosinolates, with lower levels of HGBS, NGBS, and MGBS in most genotypes in this cluster, while GBS showed no cluster-specific pattern. The first cluster included lines 8, 11, and 52, and hybrids 2, 3, 6, 7, 8, and Ambrosia.

The larger cluster contained the remaining genotypes, all characterized by below-average concentrations of 4C glucosinolates. Additionally, genotypes with the highest concentrations of SIN and GBN (3C aliphatic GSLs) were exclusively found within this cluster. The same was true for three indolyl glucosinolates: HGBS, NGBS, and MGBS. The major indolyl glucosinolate GBS again showed no clear pattern.

**Figure 4:**
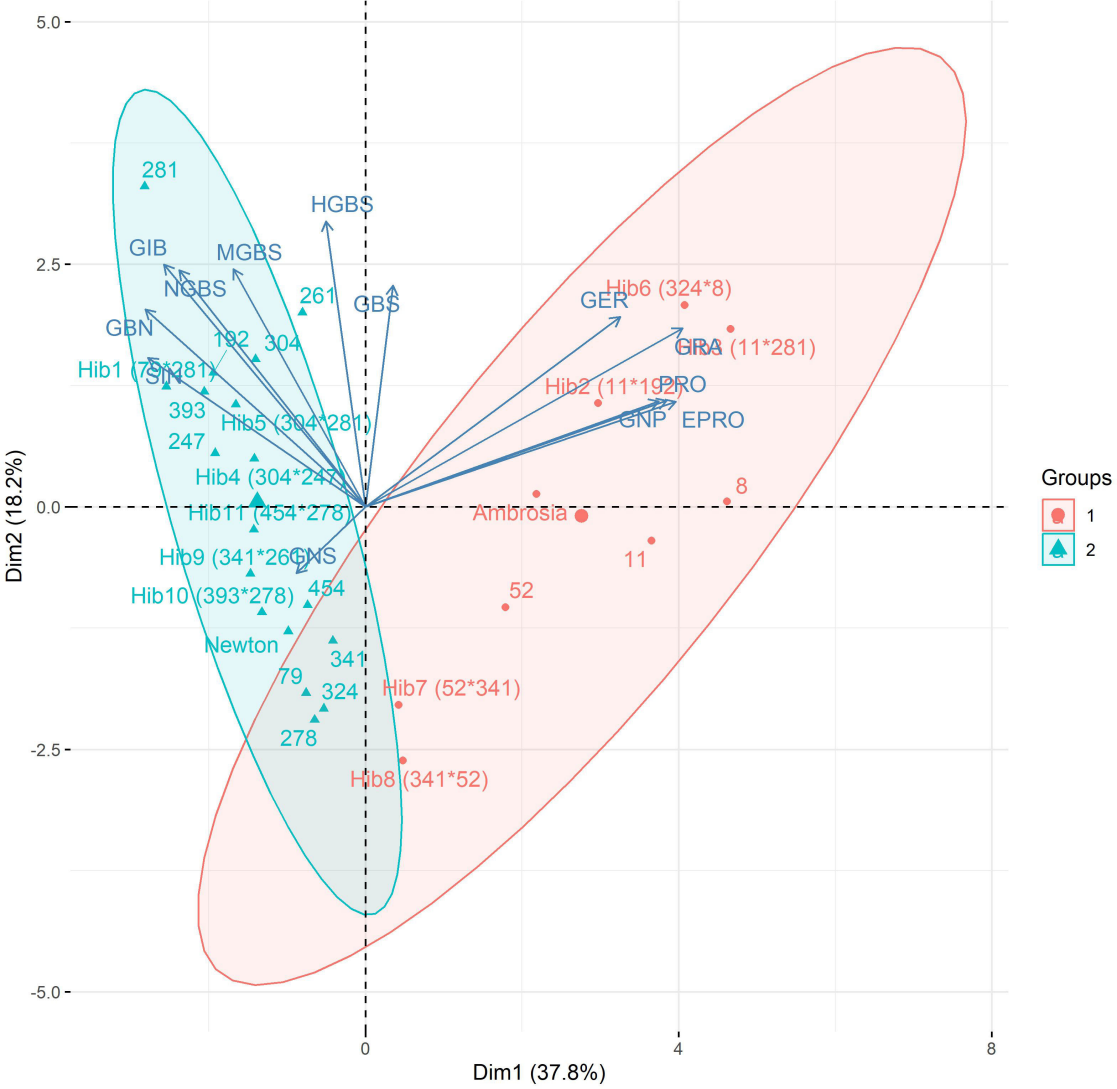
Principal component analysis (PCA) biplot based on glucosinolate profiles of cabbage genotypes. Glucosinolate loadings are shown as vectors, with ellipses indicating genotype clusters.

Patterns identified through clustering were further supported by principal component analysis (PCA). The first principal component (PC1) explained 37.8% of the total variance and was strongly correlated with 4C aliphatic glucosinolates (PRO, EPRO, GNP, GRA, and GER), and moderately correlated with 3C aliphatic glucosinolates (SIN, GBN, and GIB) and the indolyl NGBS (**Figure 4**). This shows that PC1 primarily differentiates genotypes based on 3C vs 4C aliphatic glucosinolate composition. The second principal component (PC2) explained 18.2% of the total variance and was moderately positively correlated with all indolyl glucosinolates, meaning that PC2 separates genotypes primarily by indolyl GSL content. The next two components explained an additional 15.5% (PC3) and 7.8% (PC4) of the variance but did not separate genotypes into clear clusters.

### 3.2 Correlation analysis among individual glucosinolates

**Figure 5:**
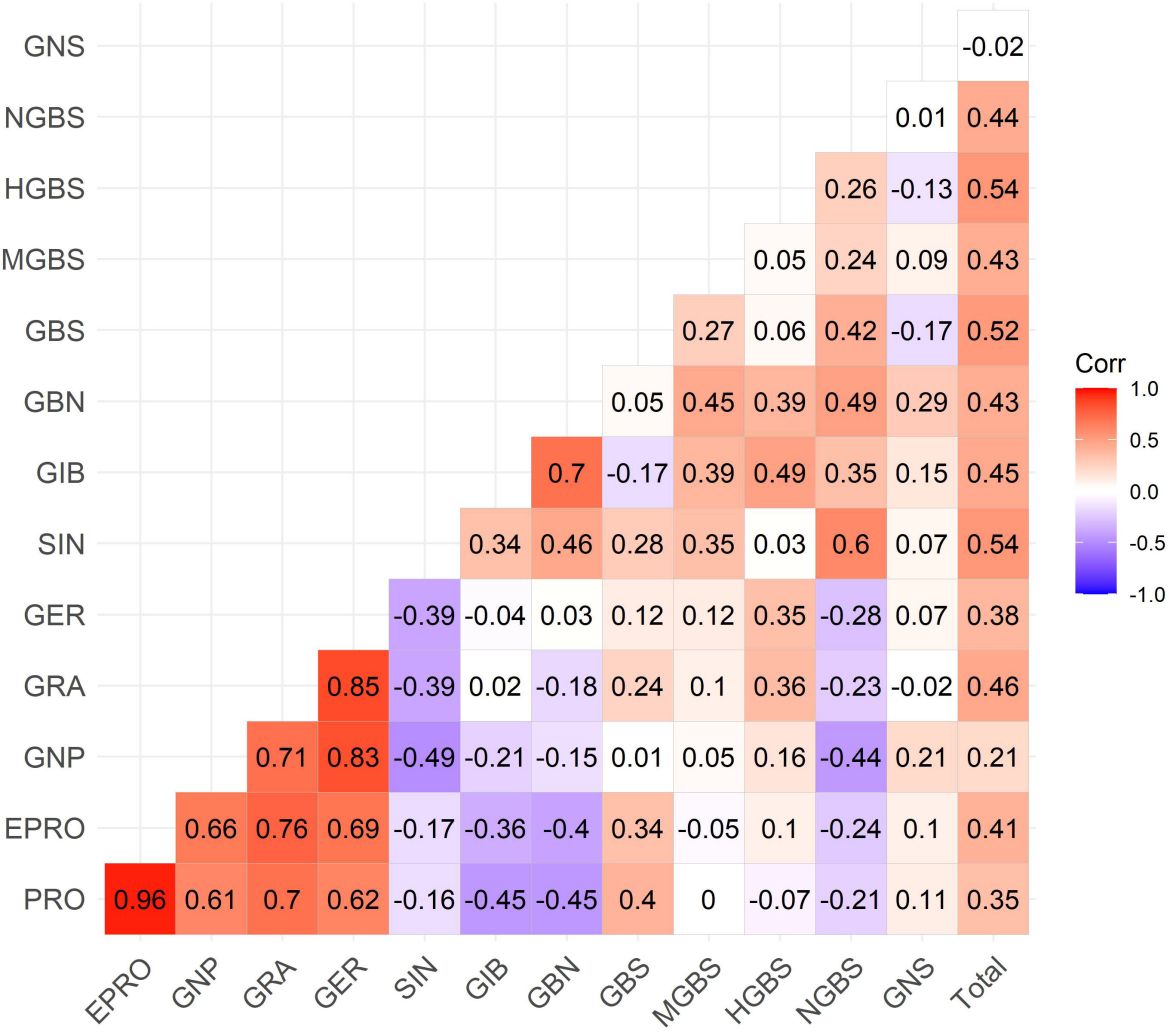
Spearman`s correlation heatmap showing pairwise relationships among the 13 identified glucosinolates. Positive correlations are shown in red, negative in blue.

To better understand the relationships among individual glucosinolates and to explore potential co-regulation within biosynthetic pathways, a Spearman correlation analysis was performed using the average concentrations of each compound across all cabbage genotypes. The results are visualized in a correlation heatmap (**Figure 5**), where the strength and direction of pairwise correlations are indicated by color intensity (red for positive, blue for negative).

Strong positive correlations were observed primarily within the 4C aliphatic GSL group derived from dihomo-methionine. All five compounds showed moderate to strong positive correlations. GER was strongly correlated with GRA (*r*_s_ = 0.85) and GNP (*r*_s_ = 0.83) and moderately correlated with PRO (*r*_s_ = 0.69) and EPRO (*r*_s_ = 0.62).

Positive correlations were also observed within the 3C aliphatic GSL group, derived from homo-methionine. GBN showed a strong correlation with GIB (*r*_s_ = 0.70) and a moderate correlation with SIN (*r*_s_ = 0.46).

Moderate to weak negative correlations were observed between most 3C and 4C aliphatic GSLs, suggesting an inverse relationship in their accumulation. The strongest negative correlations were found between GBN and PRO (*r*_s_ = −0.45), followed by GBN and EPRO (*r*_s_ = −0.34), GIB and PRO (*r*_s_ = −0.39), and GIB and EPRO (*r*_s_ = −0.36). SIN also showed moderate negative correlations with several 4C glucosinolates: GER (*r*_s_ = −0.39), GRA (*r*_s_ = −0.39), and GNP (*r*_s_ = −0.49).

The indolyl GSLs, derived from tryptophan (GBS, MGBS, NGBS, and HGBS), showed predominantly weak positive correlations among each other, except for a moderate correlation between GBS and NGBS (*r*_s_ = 0.42).

The only aromatic GSL (GNS) showed weak or negligible correlations with all other compounds, consistent with its distinct biosynthetic origin: it is derived from phenylalanine.

Total GSL content was weekly to moderately positively correlated with all individual GSLs, except for GNS. The strongest correlations were observed with the most abundant compounds, namely SIN (*r*_s_ = 0.54), GBS (*r*_s_ = 0.52), and GIB (*r*_s_ = 0.45).

### 3.3 Mid-parent heterosis for glucosinolate content

**Figure 6:**
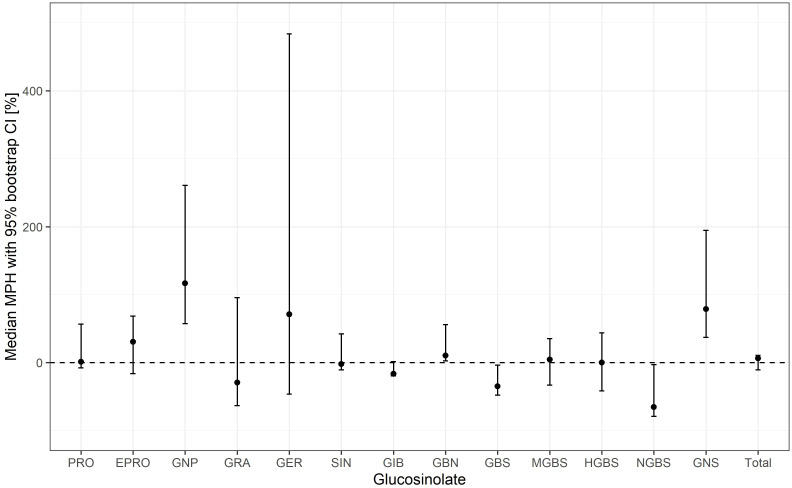
Median mid-parent heterosis (MPH) with 95% bootstrap confidence intervals (CI) of each glucosinolate.

Mid-parent heterosis (MPH) for each individual and total GSLs was calculated for 11 hybrids from our breeding program. Ranges of MPH values for each trait are summarized in **Supplementary Table S3** and in **Figure 6**.

The magnitude of MPH varied widely across GSLs and hybrids, ranging from –97.9% (NGBS in hybrid 9) to +750.6% (GER in hybrid 2). GER also exhibited the broadest range of MPH values across hybrids (from −60.1% to +750.6%) (**Supplementary Table S3**).

All values for GBS were negative (from –59.9% to –0.4%) and positive for GNP (from 10.9 to 451.0) (**Supplementary Table S3**). For other GSLs, MPV values ranged from negative to positive. Some were more skewed towards the positive range (GNS, GBN, and PRO), while others were towards the negative range (NGBS and GBS) (**Supplementary Figure S2**).

Because of differences in variability and many outliers in some hybrids, we used nonparametric bootstrap resampling to estimate 95% confidence intervals (CIs) for the median MPH for each GSL (**Figure 6**), providing a more robust assessment of whether heterosis was significantly different from zero across all hybrids. The CIs excluded zero for several GSLs. Median MPH was significantly positive for GNP, GNS, and GBN and negative for GBS and NGBS, indicating consistent heterosis for these traits.

**Figure 7:**
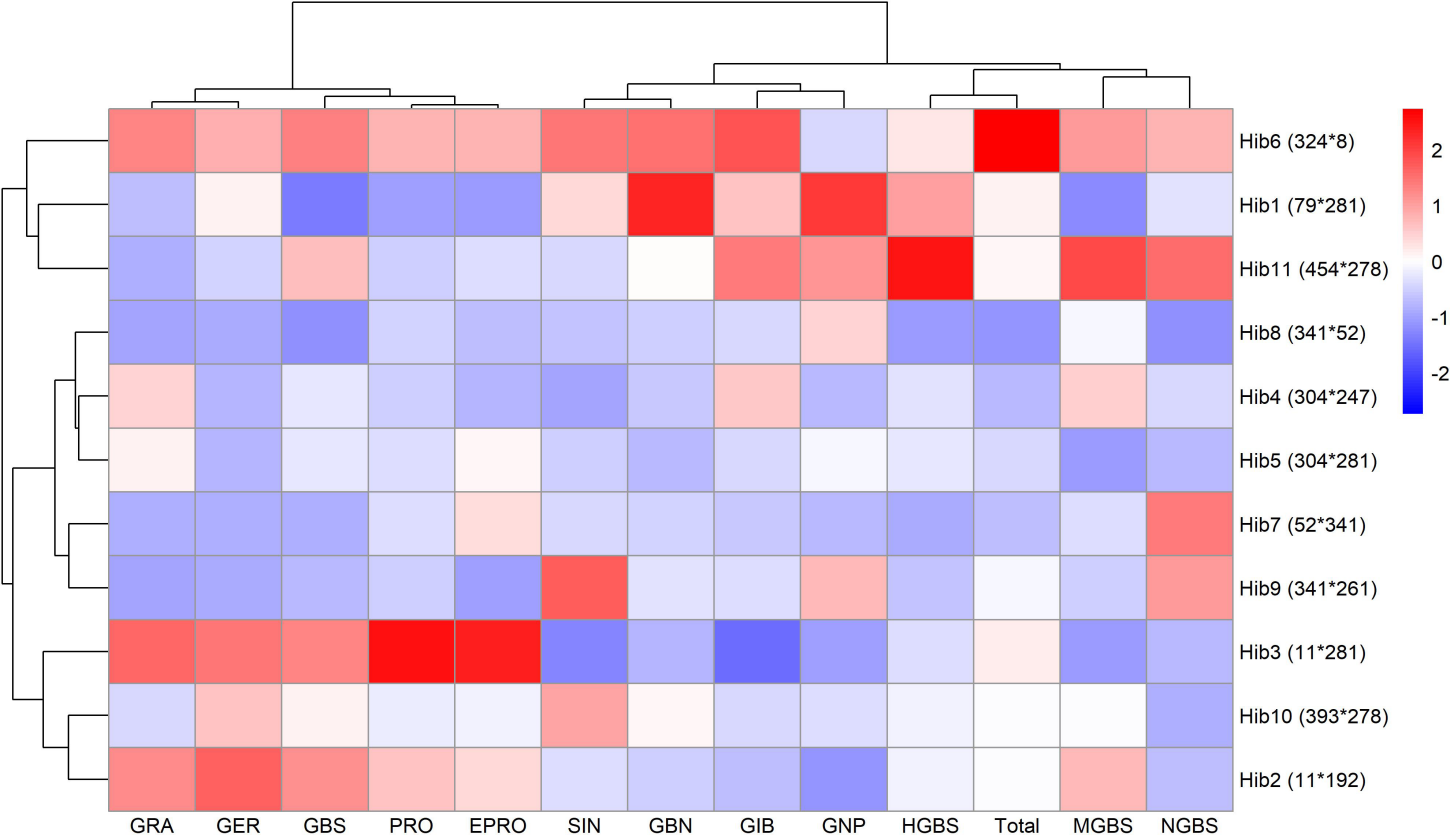
Heatmap of standardized mid-parent heterosis (MPH) of individual and total glucosinolates for each hybrid. Values above the mean are in red and values below the mean are in blue.

To better visualize heterosis patterns across hybrids, a heatmap (**Figure 7**) was generated, revealing some distinct patterns. For example, line 11, used as a parent in hybrids 2 and 3, appeared to contribute to relatively higher MPH in GRA, GER, PRO, EPRO, and GBS. Hybrid 6 displayed above-average MPH values for all glucosinolates except GNP and notably had the highest MPH for total glucosinolates.

### 3.4 Correlation between parental lines and hybrid glucosinolate content

**Figure 8:**
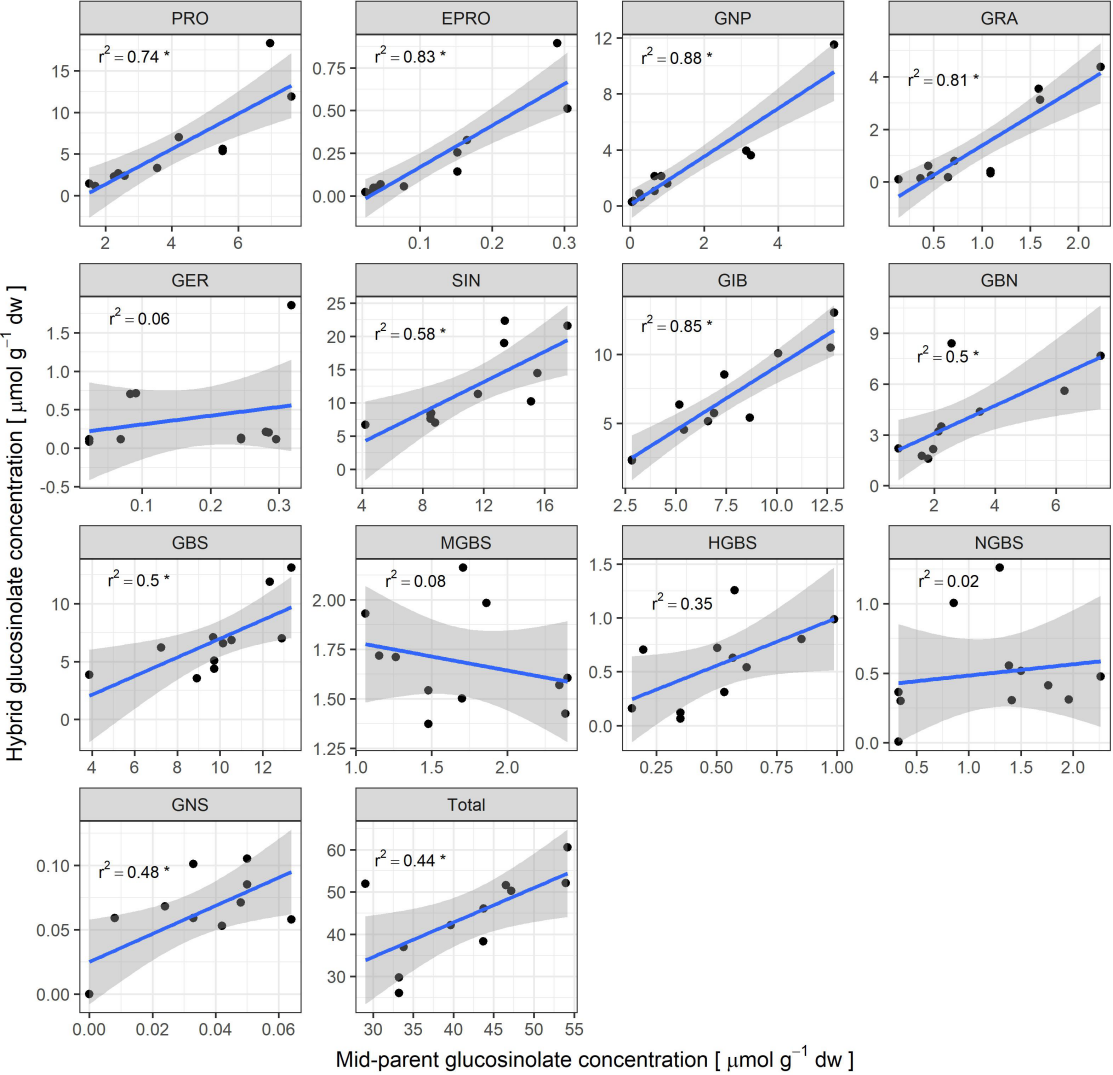
Scatter plots with linear model predictions for hybrid concentrations in dependence on mid-parent values (MPV) with 95% confidence intervals for each individual glucosinolate and total glucosinolate content. The asterisk next to r^2^ value denotes the statistically significant linear model (p < 0.05).

To assess whether parental values of glucosinolate content can be used to predict hybrid values, linear modelling was employed for individual and total GSL content (**Figure 8**).

Statistically significant linear regression models (p < 0.05) were detected for all aliphatic glucosinolates except GER. The highest proportions of variability explained were observed for GNP (r² = 0.88), GIB (r² = 0.85), EPRO (r² = 0.83), and GRA (r² = 0.81). Models for the remaining aliphatic glucosinolates accounted for lower proportions of variability, with the lowest for GBN (r² = 0.50). Among indolyl glucosinolates, only GBS showed a statistically significant model (r² = 0.50). Although the GNS model was statistically significant, the association was strongly influenced by two hybrids that lacked GNS, and exclusion of these outliers rendered the model non-significant.

### 3.5 Agronomic traits variation and heterosis analysis

In parallel with our chemical analyses, we measured five key agronomic traits—head weight (HW), head diameter (HD), head height (HH), inner‐core length (ICL), and head compactness (HC)—in the same set of 25 genotypes. Mean values with standard errors (three to four replicates per genotype) for all traits in individual genotypes are provided in **Supplementary Table S4**.

**Figure 9:**
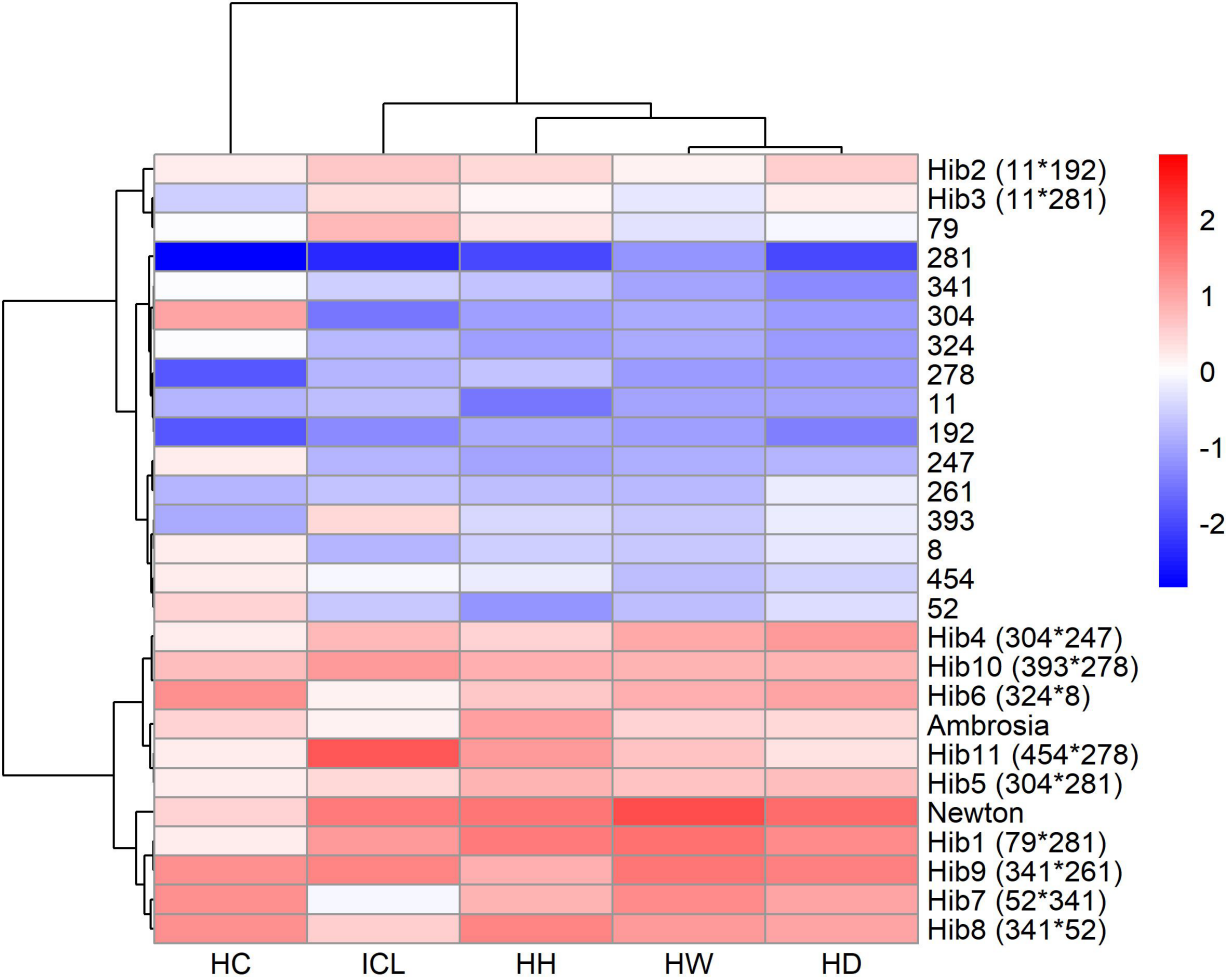
Heatmap of standardized agricultural traits—head weight (HW), head diameter (HD), head height (HH), inner‐core length (ICL), and head compactness (HC)—in all genotypes. Values above the mean are in red and values below the mean are in blue.

Hierarchical clustering of the standardized trait means (**Figure 9**) yielded two clear genotype groups. All DH lines formed a single cluster, characterized by below-average values across all traits. Almost all hybrids formed the second cluster, each exhibiting above-average trait values. A two-sample t-test with Bonferroni correction confirmed that hybrids outperformed lines for all five traits (p < 0.001). Interestingly, hybrids 2 and 3 clustered with lines, primarily because of their lower head compactness and weight. Principal component analysis (**Supplementary Figure S3**) supported these groupings: the first component (PC1, explaining 83.0% of variance) loaded heavily on HW, HD, HH, and ICL, while the second component (PC2, 10.5%) reflected variation mostly in ICL.

We also calculated mid-parent heterosis (MPH) for each trait in our 11 hybrids. All five traits exhibited positive MPH trends, again confirming hybrid vigor. The highest mean heterosis was observed for head weight (473.8%) and the lowest for head compactness (40.6%) (**Supplementary Figure S4**). Mean MPH with standard error, min, and max values is shown in **Supplementary Table S5**.

## 4 Discussion

This study aimed to characterize glucosinolate composition, variation, and correlations in cabbage inbred lines and their derived hybrids. We demonstrated substantial variation in glucosinolate profiles and content, distinct genotype groupings based on hierarchical clustering, substantial heterotic effects, and strong correlations between the glucosinolate values of parental lines and their respective hybrids.

### 4.1 Glucosinolate content and variation in cabbage genotypes

In this study, we detected 13 glucosinolates in 25 cabbage genotypes, with total concentrations ranging from 19.7 µmol g^−1^ dw to 67.8 µmol g^−1^ dw, highlighting a strong genotype effect on glucosinolate accumulation (Choi et al., 2014; Bhandari et al., 2020; Oloyede et al., 2021). This range was generally consistent with previous reports (Radovich et al., 2005; Bhandari et al., 2015; Robin et al., 2016) and reflects the broad genetic diversity of our germplasm, which was derived from commercial hybrids, local landraces, and their crosses (**Supplementary Table S1**). Such diversity is essential in hybrid breeding, as genetically distinct parental lines are needed to exploit heterosis and achieve superior offspring performance (Acquaah, 2012).

Previous studies have reported both lower (Charron et al., 2005; Kusznierewicz et al., 2008; Yi et al., 2015; Bhandari et al., 2020) and higher (Oloyede et al., 2021) total glucosinolate concentrations. These discrepancies are likely due to variation in genetic background, environmental conditions (Cartea et al., 2008), tissue selection (Kabouw et al., 2010), and methodological approaches to extraction (Ishida et al., 2011), desulfation (Doheny-Adams et al., 2017) and quantification (Lee et al., 2013). In our study, optimized extraction and analytical protocols ensured maximal recovery and consistent detection of all major glucosinolates typically found in cabbage. We did not detect two rarely reported 5-carbon aliphatics (glucoalyssin and glucobrassicanapin), which have previously been found only at trace levels (Cartea et al., 2008; Yi et al., 2015). Their absence may reflect genetic differences, extremely low abundance, or the lack of enzymes required for their precursor (trihomo-methionine) synthesis in cabbage (Harun et al., 2020).

Consistent with earlier findings (Choi et al., 2014; Bhandari et al., 2020), aliphatic glucosinolates were the dominant class in our study, followed by indolyl GSLs, with GBS, SIN, GIB, and PRO as the most abundant compounds. This stability across studies suggests that glucosinolate biosynthesis patterns in cabbage are evolutionarily conserved, likely due to their central role in plant defense (Variyar et al., 2014).

Hierarchical clustering of 25 genotypes based on glucosinolate profiles revealed two main groups (**Figure 3**): one with higher 4C (PRO, EPRO, GNP, GRA, GER) and lower 3C aliphatic GSLs (SIN, GIB, GBN), and the other showing the opposite trend. Principal component analysis confirmed this pattern (**Figure 4**), with PC1 separating genotypes by 3C vs. 4C aliphatic content, and PC2 differentiating them by indolyl glucosinolate levels. These patterns are broadly consistent with Bhandari et al. (2020), who profiled 146 cabbage accessions and similarly reported genotype separation primarily along an aliphatic–indolyl gradient, with GBS, SIN, GIB, and PRO as key contributors to variation. Interestingly, reciprocal hybrids 6 and 7, which share the same parental lines but with reversed maternal and paternal roles, exhibited nearly identical glucosinolate compositions and clustered closely on the PCA biplot. This suggests that, at least for glucosinolate-related breeding objectives, there may be no advantage in prioritizing one parental line over the other as the female parent.

A closer inspection of the hierarchical clustering heatmap revealed that genotypes within each cluster were not randomly distributed. In the smaller cluster, three parental lines (8, 11, and 52) were present, and all hybrids within this cluster had one of these lines as a parent. Similar associations were also observed in the second cluster. In addition, genetic siblings (genotypes sharing one parent) were consistently found within the same cluster, suggesting that more closely related genotypes tend to exhibit more similar glucosinolate profiles.

In contrast to the heatmap, the PCA biplot more clearly illustrated these relationships, as most hybrids clustered near their respective parental lines, and genetic siblings were almost exclusively positioned near one another. Furthermore, when considering the donor genotypes of the DH lines (**Supplementary Table S1**), lines derived from the same donor genotypes consistently clustered together based on the GSL profiles, providing additional support for the link between genetic relatedness and glucosinolate profiles.

Based on a comprehensive literature review, this study was among the first to explore glucosinolate diversity in cabbage genotypes using hierarchical clustering, and we demonstrated that this method is effective for identifying genotypes with similar glucosinolate profiles.

### 4.2 The role of glucosinolates in human health and plant defense

Selecting genotypes with the right GSL profile is crucial, as individual compounds play distinct roles in both human health and plant defense. From a human health perspective, numerous *in vitro* and *in vivo* studies have shown that GSL hydrolysis products, particularly isothiocyanates (ITCs), exert anticancer effects through multiple mechanisms (Prieto et al., 2019). Glucosinolates detected in our study, including SIN (Liu et al., 2018), GIB (Gong et al., 2021), GBN (Zhang et al., 2023), and GBS (Singh et al., 2021) have been linked to these benefits. Sulforaphane, derived from GRA, is among the most extensively studied, with substantial evidence supporting its anticancer properties (Kamal et al., 2020).

In plants, glucosinolates contribute to defense against herbivores and pathogens (Chhajed et al., 2020). Their breakdown products can slow growth, deter feeding, and reduce the fitness of generalist insects (Hopkins et al., 2009), although specialists may avoid toxicity through enzymatic detoxification or rapid excretion (Ratzka et al., 2002). For example, Robin et al. (2017) found that cabbage genotypes with higher GBS, GIB, and GBN and lower HGBS, GER, PRO, and GRA were least preferred by the diamondback moth (*Plutella xylostella* L.).

High GSL content is also positively associated with resistance to various pathogens (Liu et al., 2021). Infections by fungi such as *Sclerotinia sclerotiorum* (Abuyusuf et al., 2018a) *Mycosphaerella brassicicola* (Abuyusuf et al., 2018b) and *Leptosphaeria maculans* (Robin et al., 2020) induce accumulation of several compounds, with GBS and GBN consistently elevated. Similar trends occurred during bacterial infection by *Xanthomonas campestris* pv. *campestris* (Rubel et al., 2020), which increased eight glucosinolates.

Overall, glucosinolate profiling offers a valuable approach for selecting cabbage genotypes with both nutritional and defensive advantages, which could be selected for health benefits, general insect/pathogen resistance, or resistance to cabbage specialist insects. Breeding programs should recognize that glucosinolates beneficial for human health may not overlap with those providing pest or pathogen resistance, and that defensive profiles are often pathogen-specific. Their organoleptic effects must also be considered. Volatile degradation products of GSLs, particularly isothiocyanates, contribute to pungency, bitterness, and sulfurous aroma, with compounds such as SIN, PRO, GBS, and GNS most often linked to bitter taste (Prieto et al., 2019). However, the relationship between GSLs and sensory traits is complex and likely involves synergistic interactions with other phytochemicals, such as phenolic compounds (Cartea and Velasco, 2008). For this reason, new varieties (especially those bred for higher GSL content) should also be evaluated for sensory quality, since consumer preference ultimately determines their acceptance.

### 4.3 Glucosinolate correlation analysis

We performed correlation analysis (**Figure 5**) to examine glucosinolate co-accumulation patterns. Strong positive correlations were observed within the 4C group (PRO, EPRO, GNP, GER, and GRA). These relationships mirrored the metabolic pathway, where GER is a precursor to GRA, which is further converted to GNP and finally to PRO and EPRO (Harun et al., 2020). A similar trend (a decline in correlation strength along the biosynthetic sequence) was also found in the 3C group (GBN, GIB, and SIN).

Negative correlations between 3C and 4C aliphatic GSLs suggest a metabolic trade-off, likely due to competition for methionine-derived precursors. Both groups require methionine to form homo- and dihomo-methionine, a process dependent on methylthioalkylmalate synthase (MAM) (Li et al., 2020).

The strongest correlations with total glucosinolates were for the most abundant compounds, namely SIN (*r*_s_ = 0.54), GBS (*r*_s_ = 0.52), and GIB (*r*_s_ = 0.45), indicating their major contribution to overall variation. Similar patterns were reported by (Bhandari et al., 2015, 2020), although correlations were weaker, possibly due to greater genetic variability in their larger sample set (146 genotypes).

These correlation patterns not only support the biosynthetic relationships among structurally related glucosinolates but also align with the clustering and PCA results, in which groups of genotypes were separated primarily by their 3C and 4C aliphatic GSL profiles. These insights are also relevant for breeding, as strongly co-regulated compounds may be co-selected or manipulated together to achieve desired profiles in future cabbage cultivars.

### 4.4 Mid-parent heterosis

Our results show that heterosis for GSLs varies widely in both magnitude and direction, depending on the compound and specific parental genotype combinations. Two notable exceptions were observed: all MPH values for GBS were negative (–59.9% to –0.4%), whereas all GNP values were positive (10.9% to 451.0%). This variability of MPH is consistent with the findings of Bajpai et al. (2019) in *B. juncea*. Similarly, in cauliflower (*B. oleracea* var. *botrytis*), Dey et al. (2014) found large variation in MPH for vitamins and antioxidant pigments, further confirming that heterosis for secondary metabolites is not uniform across traits or genotypes and can be positive or negative.

As shown in **Supplementary Table S3** and **Figure 6**, GER exhibited the broadest range of MPH values (from −60.1% to +750.6%), reflecting a strong influence of specific parental combinations. The extreme positive MPH values observed for GER (and other GSLs) can be largely attributed to differences in parental GSL concentrations. In particular, very high positive MPH values occurred in hybrids in which both parents exhibited extremely low baseline GSL levels. In such cases, even moderate increases in hybrid GSL concentrations result in disproportionally large MPH values, as MPH is calculated as a relative change. In addition, genetic factors likely contribute to this variability. Given that glucosinolate biosynthesis is governed by numerous genes and regulated by diverse transcription factors (Wang et al., 2023), different parental combinations can produce diverse allelic configurations in hybrids. These, in turn, can lead to variable expression patterns shaped by a complex interplay of additive and non-additive genetic effects.

An interesting observation was made for GNS in hybrids derived from parental lines lacking this compound. In hybrids 1 and 2, both parents lacked GNS, and the hybrid progeny also failed to accumulate it. This is a strong indicator that, in these cases, both parental lines carry a nonfunctional allele of the same gene in the GNS biosynthetic pathway, which results in hybrids inheriting no functional copy of the gene and consequently lacking GNS. In contrast, in hybrids 2, 5, 6, and 9, in which only one parent lacked GNS, the hybrids were still capable of producing this compound. A plausible explanation is that the parental lines carry nonfunctional alleles for different genes, so that the hybrid inherits one functional copy of the relevant biosynthetic gene, thereby restoring GNS production. Similar cabbage inbred lines completely lacking certain glucosinolates have also been reported by Robin et al. (2016).

In our study, the median MPH was significantly positive for GNP, GNS, and GBN, and significantly negative for GBS and NGBS, indicating consistent heterosis for these traits. From a breeding perspective, such consistency suggests that improvement of these glucosinolates through heterosis-based strategies should be more predictable.

### 4.5 Correlation between parental lines and hybrid glucosinolate content

Information on parental inbred lines is valuable for hybrid breeding, but only if it reliably predicts hybrid performance, thereby substantially reducing the need for extensive testcrossing and expensive field trials. We examined the relationship between mid-parent values (MPV) and hybrid concentrations for individual and total GSLs to evaluate the potential of parental phenotyping as a predictive tool in GSL breeding.

Our analysis revealed statistically significant linear regression models for most aliphatic glucosinolates, with the percentage of variability explained ranging from 88% for GNP (r² = 0.88) to 50% for GBN (r² = 0.50). High r² values indicate a strong influence of parental genotypes on hybrid performance and are consistent with high heritability estimates (Alvarez Prado et al., 2013). In contrast, among indolyl glucosinolates, only the model for GBS was significant, explaining 50% of the variability (r² = 0.50). This divergence between aliphatic and indolyl glucosinolates likely reflects differences in their genetic architecture and/or regulation.

The stronger parent–hybrid correlations observed for aliphatic glucosinolates may be linked to their relatively simpler and more coordinated transcriptional control, where a small group of MYB transcription factors (MYB28, MYB29, and MYB76) act as strong positive regulators of aliphatic GSL biosynthesis (Harun et al., 2020) and manipulation of these regulators can substantially upregulate aliphatic GSL biosynthetic genes (Qin et al., 2023). In contrast, weaker correlations for indolyl glucosinolates likely reflect more complex and environmentally responsive regulation. Although indolyl GSL biosynthesis is positively regulated by MYB2, MYB3, and MYB4 (Schweizer et al., 2013), other key regulators are directly linked to hormone signaling pathways. In particular, MYB34 regulates indolyl GSL biosynthesis in coordination with abscisic and jasmonic acid, MYB51 with salicylic acid and ethylene, and MYB122 with jasmonic acid and ethylene (Frerigmann et al., 2016). Because plant hormones are highly sensitive to environmental conditions (Kumari et al., 2024), this hormone-dependent regulation of indolyl GSLs could explain why crosses between different parental genotypes show more complex and environmentally dependent inheritance patterns, thereby reducing the predictability of hybrid performance for these compounds.

Several studies in cabbage have also reported associations between glucosinolate biosynthetic gene expression levels and glucosinolate accumulation. Robin et al. (2016) showed that cabbage inbred lines with higher expression of *ST5b* accumulated higher levels of aliphatic glucosinolates (GRA, GNP, and SIN), while higher expression of *MYB34* and *CYP81F1* was associated with increased NGBS content. Similarly, Abuyusuf et al. (2018b) linked white mold resistance to increased GIB and GBS and reported positive associations between GIB content and expression of *ST5b/ST5c*, as well as between GBS content and expression of *ST5a* genes, indicating pathogen-induced transcriptional activation of GSL biosynthesis and accumulation. In black rot infected cabbage, Rubel et al. (2020) reported positive correlations between aliphatic GSL compounds and expression of *ST5c* and *AOP2*, and between indolyl GSL compounds and expression of *MYB34*, *MYB122*, and several *CYP81F* genes (Rubel et al., 2020), further supporting the link between gene expression and GSL accumulation under biotic stress. At the genomic level, Wang et al. (2023) identified 193 cabbage orthologs of *A. thaliana* GSL biosynthetic genes and found that the expression of most genes was significantly genotype-dependent. They also identified numerous genes whose expression was altered after plant hormone spraying. For instance, methyl jasmonate upregulated certain side chain extension genes (*BoIPMILSU1–1* and *BoBCAT-3-1*) and core structure construction genes (*BoCYP83A1* and *BoST5C-1*), while ethylene downregulated certain side chain extension genes (*BoIPMILSU1-1*, *BoCYP79B2-1*, and *BoMAMI-1*) and transcription factors for both aliphatic and indolyl GSL (*BoMYB28-1*, *BoMYB34-1*, *BoMYB76-1*, and *BoCYP79B2- 1*).

More generally, gene expression is shaped by complex interactions among DNA, RNA, proteins, and the environment (Signor and Nuzhdin, 2018) and combining different parental genotypes can further increase this complexity, limiting the predictive value of parental gene expression for hybrid phenotypes (Wang et al., 2024) For breeding, phenotyping therefore remains particularly valuable because it captures the net outcome of all underlying genetic and regulatory contributions. Accordingly, the strong, statistically significant regression models for most aliphatic glucosinolates in our study indicate that hybrid performance can be reliably predicted from parental phenotypes, enabling targeted parent selection and reducing the need for extensive test crosses. For GBS, prediction from parental phenotypes may still be feasible, but with lower accuracy due to its moderate r² value. For the remaining indolyl glucosinolates, direct hybrid evaluation remains essential and integrating marker-assisted or genomic selection strategies could improve prediction accuracy.

### 4.6 Agronomic traits

Our agronomic evaluations revealed a clear separation between lines and hybrids, with all measured traits (head weight, diameter, height, inner‐core length, and compactness) significantly higher (p < 0.001) in hybrids. Accordingly, all traits exhibited positive MPH values across hybrids, confirming the consistent expression of hybrid vigor. The exceptionally high mean heterosis for head weight (473.8%) demonstrates the substantial biomass gains achievable through hybridization. Such patterns are characteristic of classical hybrid vigor, a phenomenon well-documented across crops (Labroo et al., 2021). From a breeding perspective, these results reaffirm that hybrid breeding is a highly effective strategy for improving yield in cabbage, explaining why most modern cultivars are hybrids.

## 5 Conclusion

In this study, we profiled 13 glucosinolates in cabbage doubled haploid lines and their derived hybrids, revealing substantial genotypic variation in both composition and total content. Hierarchical clustering revealed two distinct genotype groups, differentiated primarily by 3C and 4C aliphatic GSL profiles, which coincided with patterns of GSL correlations. Heterosis for GSLs varied substantially across compounds and parental combinations, with consistent positive effects for GNP, GNS, and GBN and negative effects for GBS and NGBS. Statistically significant linear regression models (p < 0.05) were observed for most aliphatic GSLs and indolyl GBS, supporting the predictive value of parental phenotyping. Agronomic traits showed uniformly positive mid-parent heterosis (MPH), with exceptionally high MPH values for head weight, confirming the advantage of hybrid breeding for yield improvement.

These findings demonstrate the potential to integrate GSL profiling of inbred lines into cabbage breeding programs to improve health-promoting and defense-related glucosinolates alongside yield. Extending this approach to other secondary metabolites, such as polyphenols, could further enhance the nutritional and defense-related value of new varieties. Future work should combine biochemical phenotyping with genomic approaches, such as QTL mapping and genome-wide association studies, to clarify the genetic basis of glucosinolate regulation, accumulation, and inheritance in cabbage and to develop marker-assisted or genomic selection strategies, particularly for traits with weaker parental–hybrid predictability. Larger studies across diverse genetic backgrounds and targeted crosses of high-GSL parental lines will be essential to confirm and generalize these findings.

For the first time in cabbage, we show that parental glucosinolate values can predict hybrid performance, providing breeders with a practical tool to select parents for developing high-yielding, nutritionally enhanced, and more pest- and disease-resistant hybrids.

## Data Availability

The raw data supporting the conclusions of this article will be made available by the authors, without undue reservation.
