# Correction: Glucosinolate variation, heterosis, and prediction of hybrid performance from parental values in white cabbage (*Brassica oleracea* var. *capitata*)

**DOI:** 10.3389/fpls.2026.1836820

**Published:** 2026-03-31

**Authors:** Primož Fabjan, Maja Mikulič Petkovšek, Damijana Kastelec, Adriana Podržaj, Katarina Rudolf Pilih

**Affiliations:** Biotechnical Faculty, University of Ljubljana, Ljubljana, Slovenia

**Keywords:** cabbage, doubled haploid lines, glucosinolates, heterosis, hybrid breeding, nutritional value

The funder “P4-0013-048 (Horticulture)” was erroneously omitted from the **Funding** statement.

The original version of this article has been updated.

