# Supplementary material for: Glucosinolate variation, heterosis, and prediction of hybrid performance from parental values in white cabbage (Brassica oleracea var. capitata)
Source: Front Plant Sci. 2026 Feb 20;17:1703515. doi: 10.3389/fpls.2026.1703515 (PMC12963295; doi:10.3389/fpls.2026.1703515)
Supplement: Supplementary file 1 [file DataSheet1.docx]

Supplementary Material

# Supplementary Figures


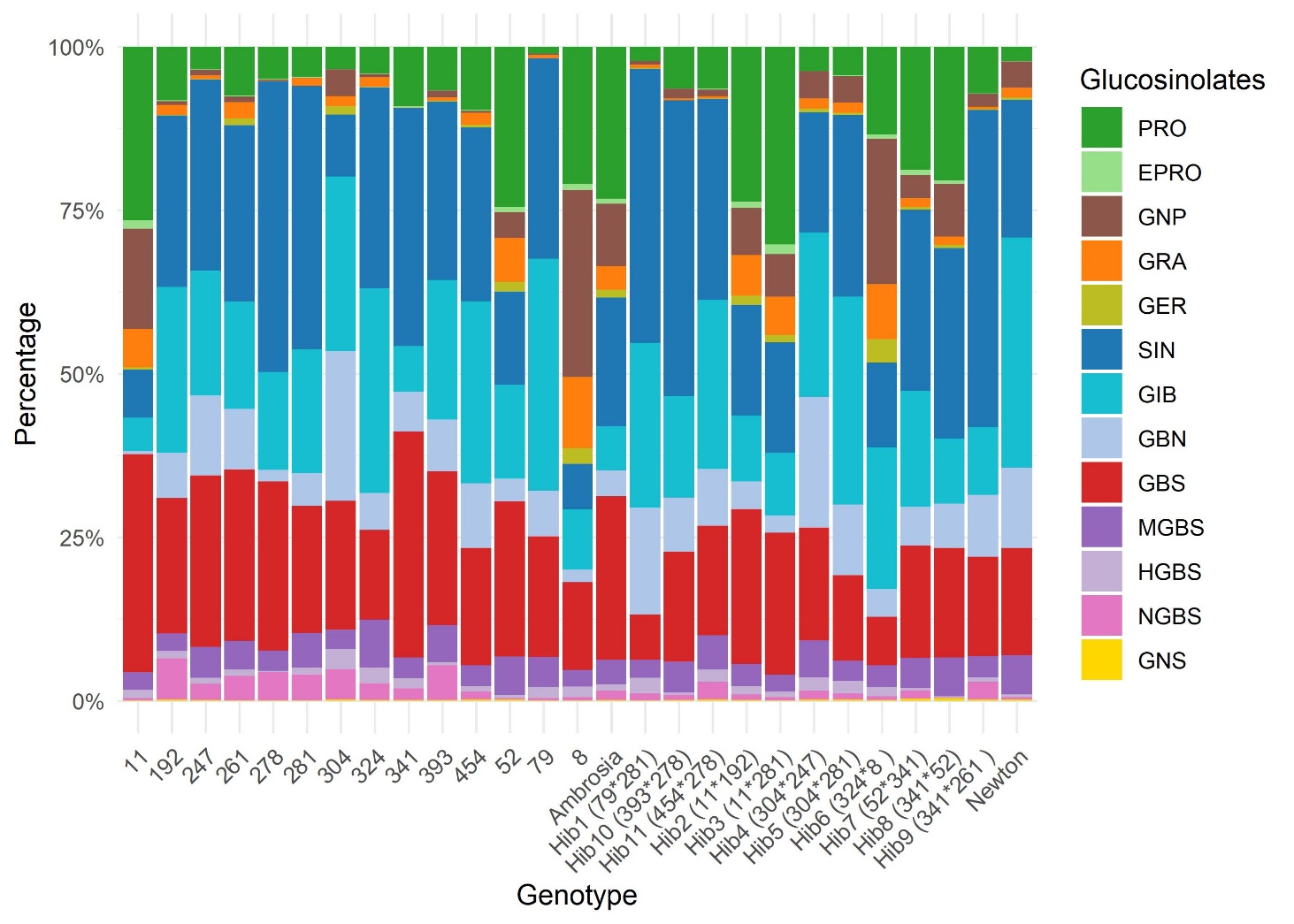


**Supplementary Figure S1.** Mean proportional glucosinolate composition of 13 individual glucosinolates for each cabbage genotype.


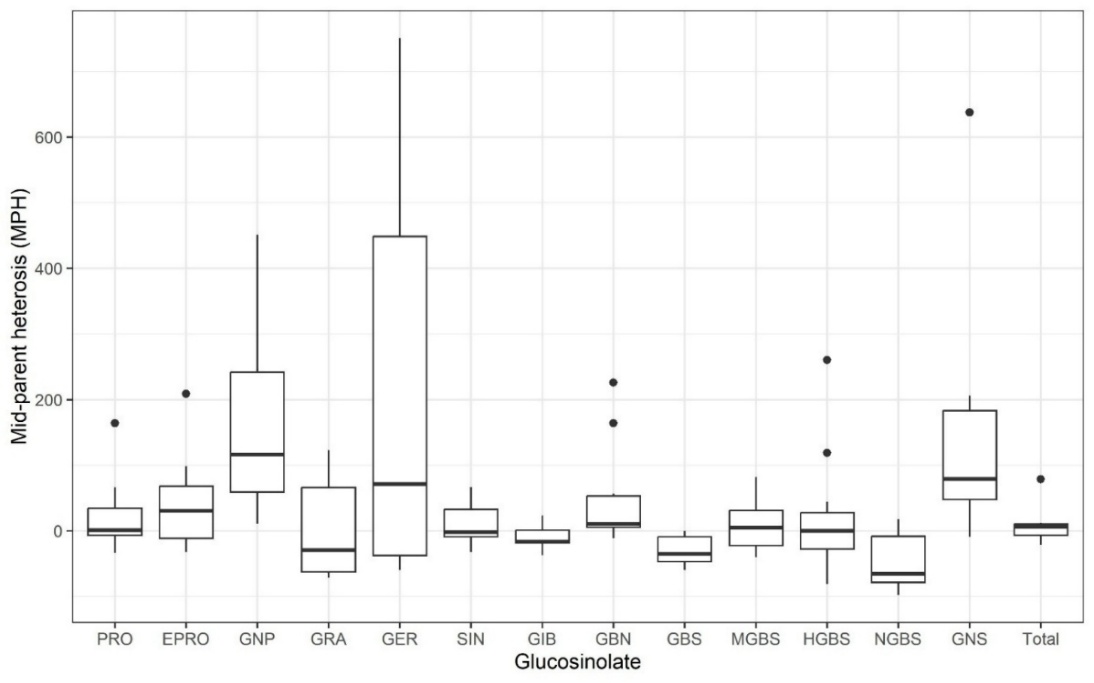


**Supplementary Figure S2**: Boxplots for mid-parent heterosis (MPH) in 11 cabbage hybrids, showing median, interquartile range (Q1–Q3), and outliers.


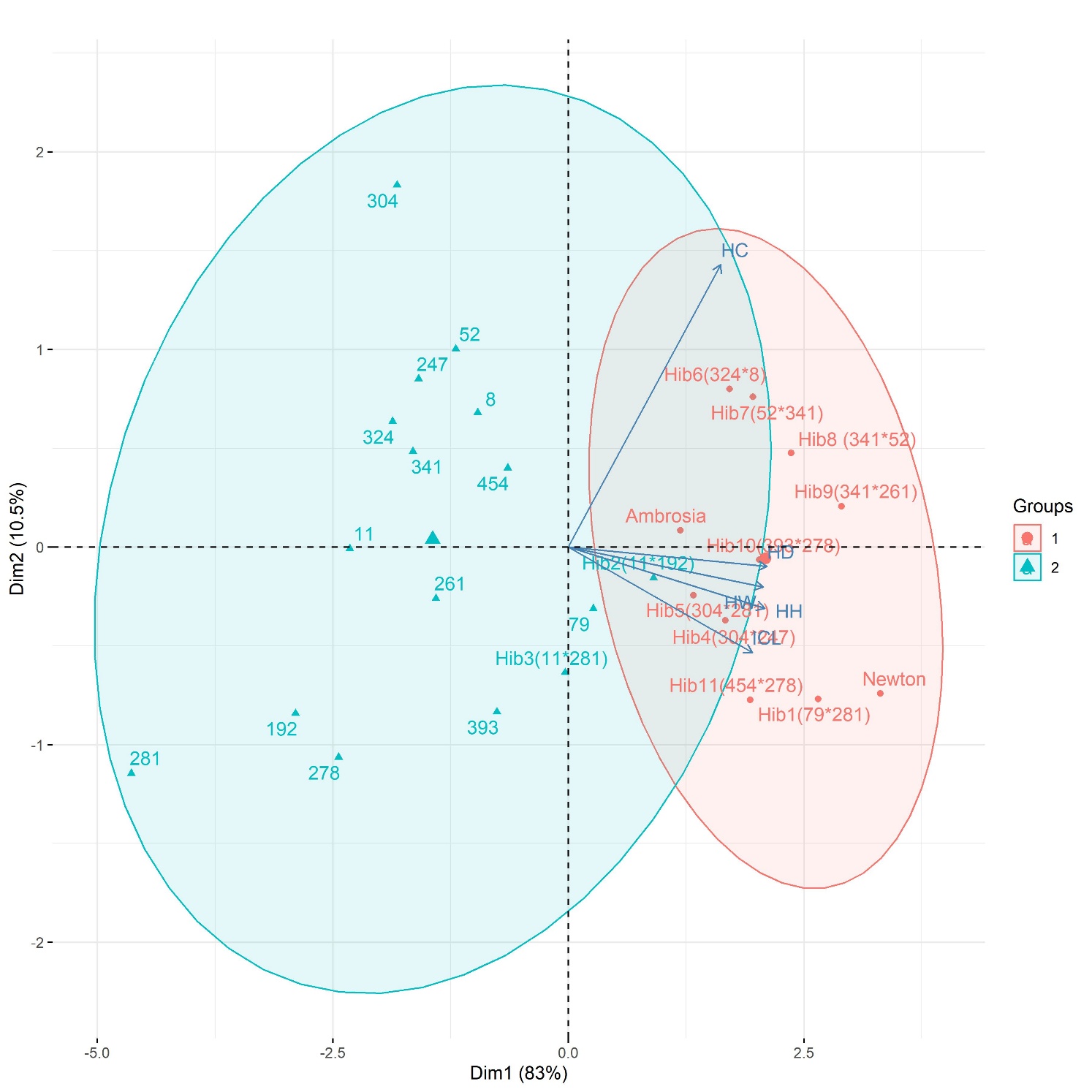


**Supplementary Figure S3**: Principal component analysis (PCA) biplot based on horticultural traits of cabbage genotypes. Traits loadings are shown as vectors, with ellipses indicating genotype clusters.


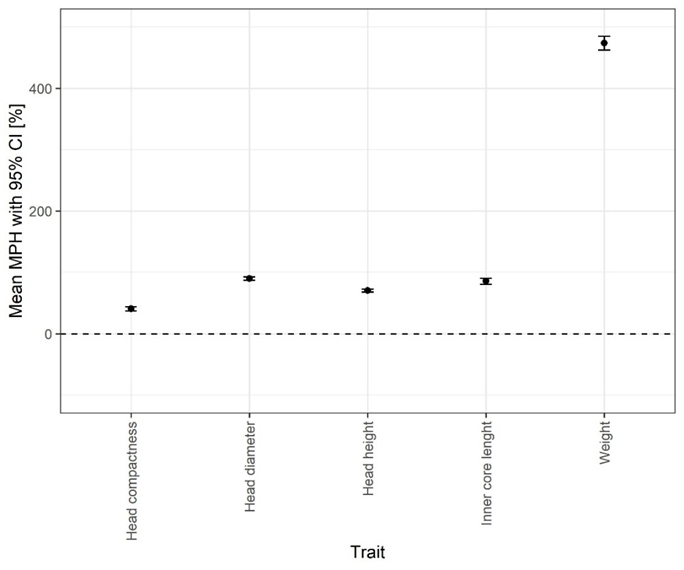


**Supplementary Figure S4**: Mean mid-parent heterosis (MPH) across all hybrids for horticultural traits, with bars showing 95% confidence intervals (CI).
